# Cellulose-based hydrogel materials: chemistry, properties and their prospective applications

**DOI:** 10.1007/s40204-018-0095-0

**Published:** 2018-09-04

**Authors:** S M Fijul Kabir, Partha P. Sikdar, B. Haque, M. A. Rahman Bhuiyan, A. Ali, M. N. Islam

**Affiliations:** 1 Department of Textiles, Apparel Design and Merchandising, Louisiana State University, Baton Rouge, LA 70803 USA; 2 Department of Textiles, Merchandising and Interiors, University of Georgia, Athens, GA 30602 USA; 3 College of Textile Engineering, University of Chittagong, Chittagong, 4331 Bangladesh; 4 Department of Textile Engineering, Dhaka University of Engineering and Technology, DUET, Gazipur, 1700 Bangladesh; 5 Department of Chemistry, Dhaka University of Engineering and Technology, DUET, Gazipur, 1700 Bangladesh

**Keywords:** Hydrogels, Cellulose, Chitin, Chitosan, Smart material, Biomedical applications

## Abstract

**Abstract:**

Hydrogels based on cellulose comprising many organic biopolymers including cellulose, chitin, and chitosan are the hydrophilic material, which can absorb and retain a huge proportion of water in the interstitial sites of their structures. These polymers feature many amazing properties such as responsiveness to pH, time, temperature, chemical species and biological conditions besides a very high-water absorption capacity. Biopolymer hydrogels can be manipulated and crafted for numerous applications leading to a tremendous boom in research during recent times in scientific communities. With the growing environmental concerns and an emergent demand, researchers throughout the globe are concentrating particularly on naturally derived hydrogels due to their biocompatibility, biodegradability and abundance. Cellulose-based hydrogels are considered as useful biocompatible materials to be used in medical devices to treat, augment or replace any tissue, organ, or help function of the body. These hydrogels also hold a great promise for applications in agricultural activity, as smart materials and some other useful industrial purposes. This review offers an overview of the recent and contemporary research regarding physiochemical properties of cellulose-based hydrogels along with their applications in multidisciplinary areas including biomedical fields such as drug delivery, tissue engineering and wound healing, healthcare and hygienic products as well as in agriculture, textiles and industrial applications as smart materials.

**Graphical abstract:**

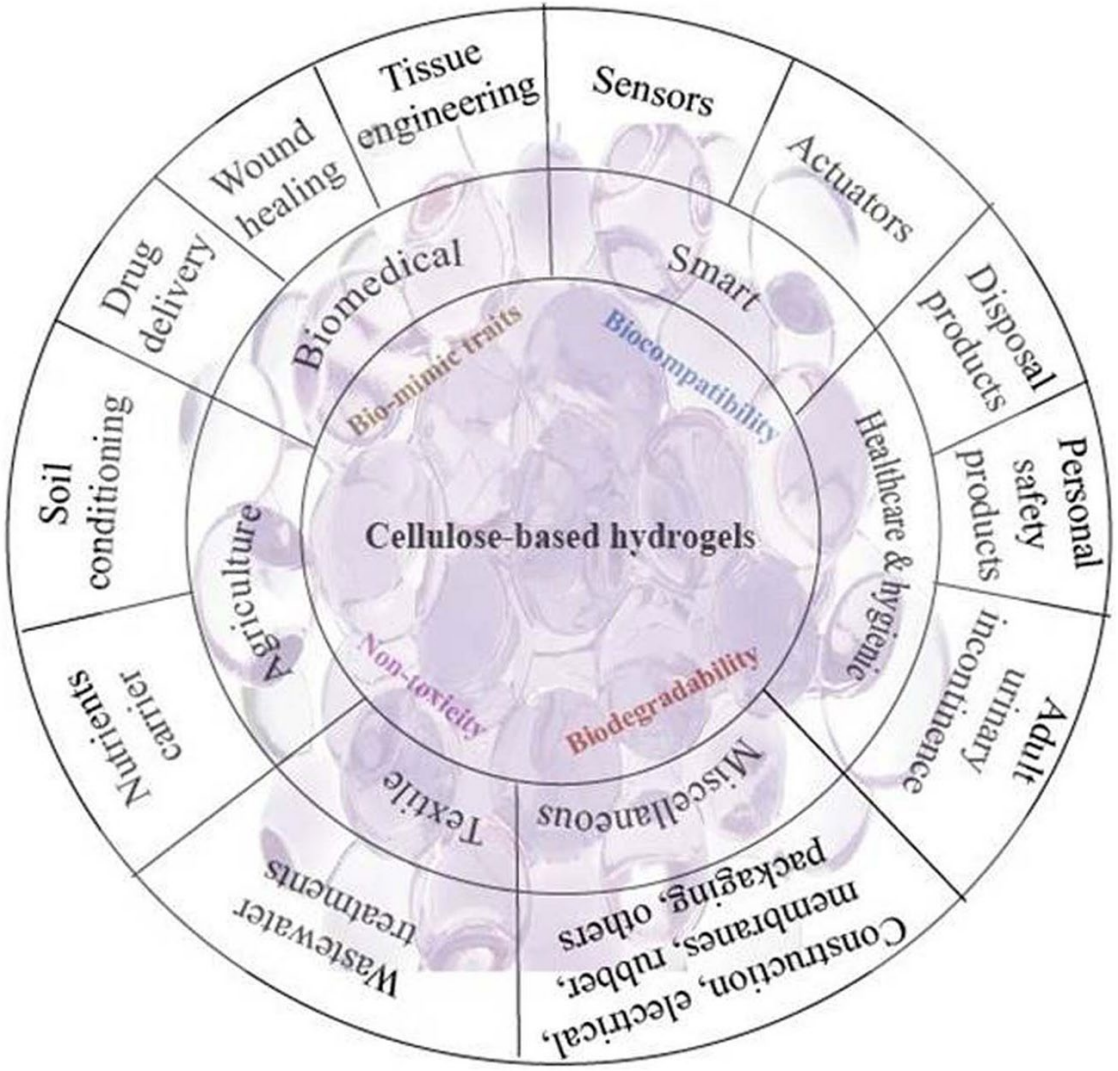

## Introduction

Hydrogels are polymeric materials with three-dimensional (3D) network structure having hydrophilic polymer chains as well as their ability to absorb and retain a large volume of water in their interstitial structures (Chai et al. 2017; Ebara et al. 2014; Yuan 2013). Upon contact with water, hydrogels continue to absorb and swell to form 3D structure due to the presence of hydrophilic groups, such as–NH_2_, –OH, –COOH, –SO_3_H in their polymer networks and osmotic pressure (Yuan 2013). The ability to hold an unaltered 3D structure during swelling is caused by physical or chemical crosslinking that also helps prevent hydrogels from dissolution in the solvent (Rizwan et al. 2017). Physical crosslinking is a temporal connection due to hydrogen bonding, hydrophobic interactions or electrostatic interactions between polar groups. On the other hand, chemical crosslinking is a permanent junction formed by covalent bonds, along with a relatively stronger ionic communication between various functional groups of introduced crosslinking agents (Ahmed 2015). Therefore, different polymeric structures including homo-polymers, linear copolymers, and block or graft copolymers are formed because of physical and/or chemical crosslinking during the polymerization process (Hoffman 2012). Besides, hydrogels are being used in various physical forms, which could be solid molded forms (soft contact lenses), pressed powder matrices (pills or capsules for oral ingestion), microparticles (as bio-adhesive carriers or wound treatments), coatings (on implants or catheters), membranes or sheets (as a reservoir in a transdermal drug delivery patch), encapsulated solids (in osmotic pumps) and liquids (that form gels upon heating or cooling) (Ebara et al. 2014).

Hydrogels are generally availed from the natural source (natural hydrogels) or synthesized through chemical reactions. Naturally sourced hydrogels often called biopolymer-based hydrogels, have some idiosyncratic attributes unlike synthetic hydrogels in terms of biocompatibility, biodegradability, non-toxicity, bio-mimic traits (Mogoşanu and Grumezescu 2014). However, some synthetic hydrogels or chemically modified hydrogels (hybrid hydrogels) find some improved features nowadays in terms of functionality, which makes them more attractive than natural hydrogels. Biopolymer-based hydrogels are sourced mainly from the plant and/or animal extract (Shen et al. 2016) as well as from cellulose, which are termed as cellulose-based (CB) hydrogels. These hydrogels can be prepared from pure and native cellulose by chemical dissolution with LiCl/dimethylacetamide (DMAc), *N*-methylmorpholine-*N*-oxide (NMMO), ionic liquids (ILs), alkali/urea (or thiourea), or by fabricating/designing with bacterial cellulose (Shen et al. 2016). Cellulose derivatives are comprised usually of either esters (e.g., cellulose acetate (CA), cellulose acetate phthalate (CAP), cellulose acetate butyrate (CAB), cellulose acetate trimellitate (CAT), hydroxypropyl methylcellulose phthalate (HPMCP)) or ethers (e.g., methylcellulose (MC), ethyl cellulose (EC), hydroxyethyl cellulose (HEC), carboxymethyl cellulose (CMC), sodium carboxymethyl cellulose (NaCMC), hydroxypropyl cellulose (HPC) and hydroxypropyl methylcellulose (HPMC)). Another recent type is composite hydrogels made up of blending composites with natural polymers, polyvinyl alcohol, polyelectrolyte complexes, interpenetrating polymer network, cellulose-inorganic hybrid hydrogels (Chang and Zhang 2011; Onofrei and Filimon 2016; Sannino et al. 2009).

**Fig. 1:**
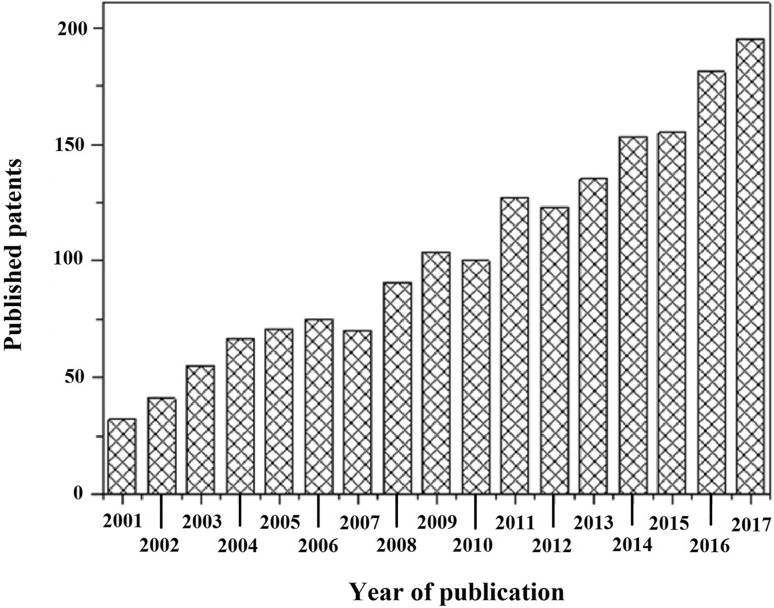
An overview of the number of published patents concerning cellulose-based hydrogels over the last decades

The appealing functionalities of CB hydrogels inspire researchers throughout the globe to develop new materials for myriad applications in various fields, such as biomedical engineering (tissue engineering, wound dressing, drug delivery system), development of smart materials (sensors, actuators), advancement in healthcare and hygienic products (diapers, napkins) along with improvement in agriculture (pesticide carriers, water reservoir, water retention). The research boom in the different array of CB hydrogels has ensued hundreds of patents and numerous research articles, which have been published in recent times (Rodrigues et al. 2014). The trend of published patents over the last decades concerning CB hydrogels and their various applications are presented in Fig. 1.

Besides, a significant number of review articles have also been published on polysaccharide and its derivatives including cellulose regarding different contexts as shown in Table 1. These review articles are mainly focused on the preparation and design of CB hydrogels along with their biomedical, agricultural and hygienic applications separately. However, to the best of our knowledge, a comprehensive review with a broad picture of CB hydrogel including their hygienic and non-hygienic applications are not discussed yet. Hence, the aim of the present review is to concentrate on the current research on biopolymer cellulose-based hydrogels as well as analyze the scope of their wide range of applications in various fields. The organization of this article is devoted to the chemical structure of cellulose-based hydrogels, highlighting the physicochemical properties of these polymers followed by applications in wide-ranging fields as well as underscoring the effects of polymer characteristics on their applications through a meticulous analysis of the contemporary and recent studies.

**Table 1:** Published review articles on different aspects of polysaccharide and its derivative-based hydrogels

Polysaccharide and its derivative-based hydrogels	Context of the review	References
Polysaccharide-based	Characterization and properties	Magnani et al. (2000)
	Release formulations	Coviello et al. (2007)
	Preparation, characterization and agricultural applications	Guilherme et al. (2015)
	Protein drug delivery applications	Chen et al. (1995)
Psyllium polysaccharide-based	Structure, synthesis, and biomedical, flocculation and water treatment applications	Thakur and Thakur (2014)
Chitosan-based	Characteristics and pharmaceutical applications	Ahmadi et al. (2015)
	Controlled, localized drug delivery applications	Bhattarai et al. (2010)
	Characteristics and pharmaceutical applications	Ahmadi et al. (2015)
	Nasal drug delivery applications	Luppi et al. (2010)
Cellulose-based	Smart swelling and controllable delivery applications	Chang et al. (2010)
	Biomedical applications	Chang and Zhang (2011)
	Preparation, and agricultural, hygienic, water treatment and biomedical applications	Ma et al. (2015)
	Biomedical and environmental applications	Onofrei and Filimon (2016)
	Design, and hygienic, agricultural and biomedical applications	Sannino et al. (2009)
Cellulose and chitin-based	Fabrication, properties, and biomedical and water treatment applications	Shen et al. (2016)
Cellulose/chitin composites	Preparation and characterization	Rodrigues et al. (2014)
Starch-based	Synthesis, and agricultural, water treatment, biomedical, electrical and construction applications	Ismail et al. (2013)
	Fabrication and biomedical applications	Zhang et al. (2005)
	Synthesis, hygienic, medical, constructional, agricultural and other applications	Athawale and Lele (2001)
Cellulose-derivatives-based	Dermal and transdermal drug delivery analysis and applications	Vlaia et al. (2016)
Carboxymethyl cellulose-based	Water treatment applications	Yang et al. (2011)

### Natural hydrogels and their derivatives

**Fig. 2:**
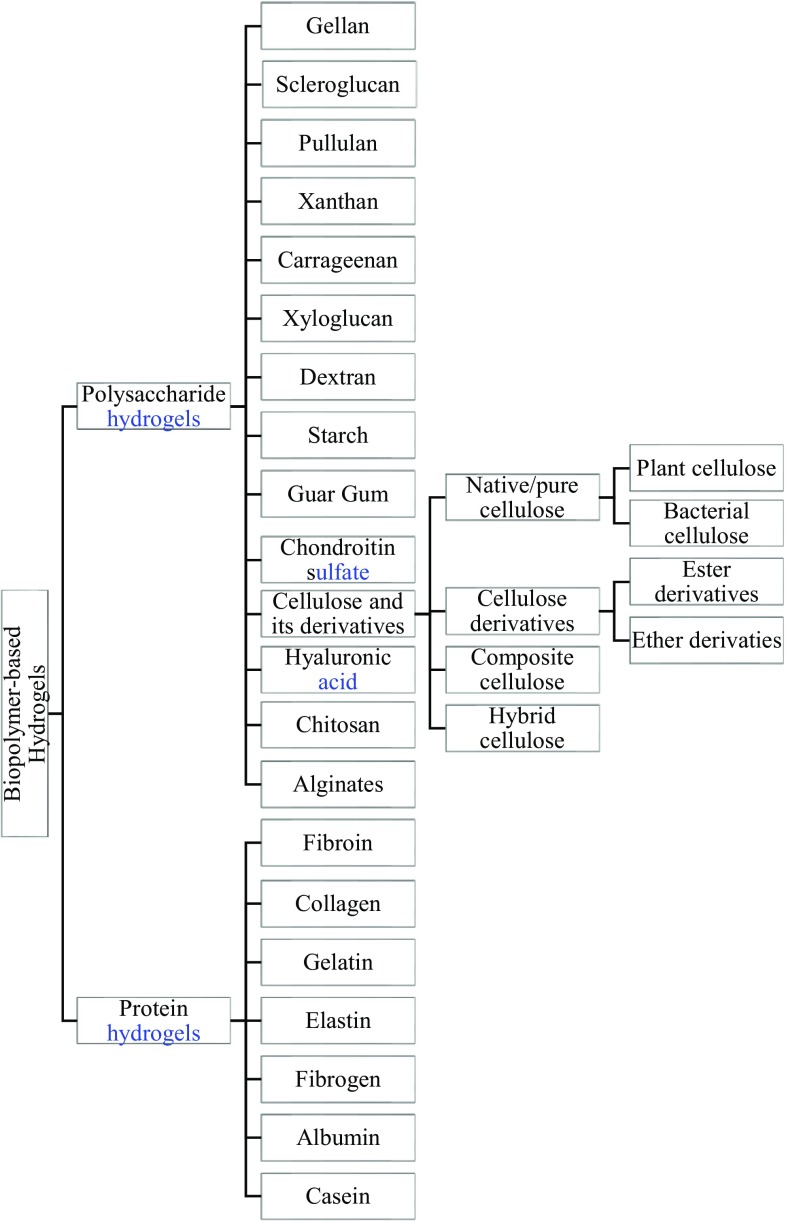
Classification of natural hydrogels based on source

Natural hydrogels are commonly derived from polysaccharide and protein derivatives (Le et al. 2017). Cellulose and its derivative based hydrogels are developed from polysaccharides (Kwiecień and Kwiecień 2018). Considering the context of the review, natural hydrogels from cellulose and its derivatives have been further classified as shown in Fig. 2.

### Native/pure cellulose-based hydrogels

**Fig. 3:**
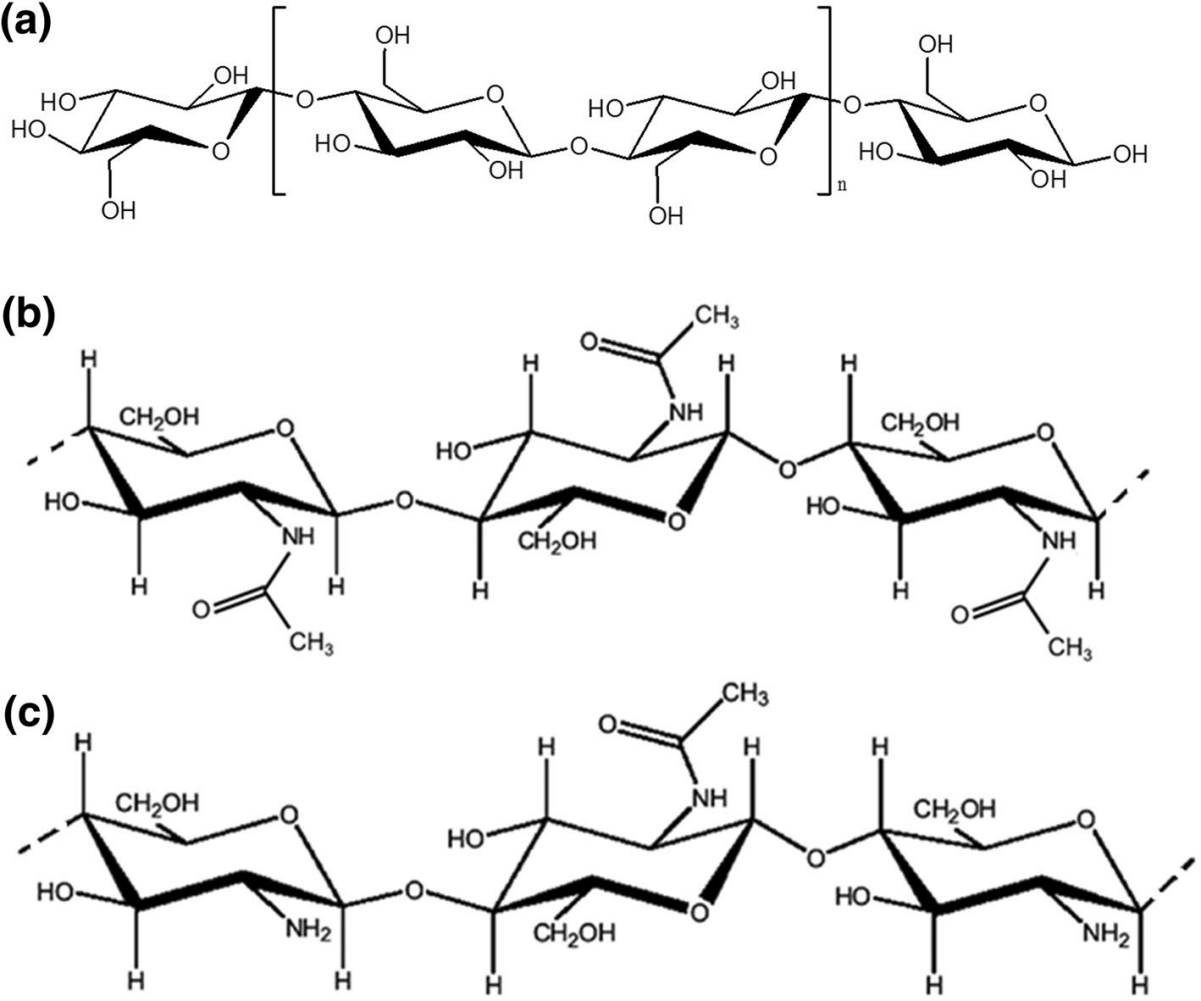
Chemical structure of **a** cellulose, **b** chitin and **c** chitosan polymer

Hydrogels based on natural cellulose can be prepared from a pure cellulose solution through physical cross-linking due to the presence of numerous hydroxyl groups, which can link polymer network through hydrogen bonding (Shen et al. 2016). Cellulose is the most abundant naturally occurring polymer of glucose (Fig. 3a), which are found in plants and natural fibers such as cotton and linen (Bhuiyan et al. 2017; Edgar et al. 2001; Islam et al. 2017). Moreover, chitin, chitosan and bacterial cellulose, comprising hydroxyl groups can also be used as alternative sources of cellulose to develop hydrogels with fascinating structures and properties (Chang and Zhang 2011). The hydroxyl group at position C-2 of cellulose chain has been replaced by an acetamide (CH_3_CONH–) group in chitin structure (Fig. 3b). If the acetamide groups of chitin are transformed into primary amino groups, they would turn into *N*-deacetylated derivatives being called chitosan (Fig. 3c) (Aranaz et al. 2009). Microbial or bacterial cellulose (BC), synthesized from bacteria (e.g., acetobacterxylinum) (Ross et al. 1991; Sannino et al. 2009), has the chemical group like plant cellulose (PC), but they have different macromolecular structures and physical properties. In both BC and PC, the glucose units are held together by 1,4-β-glucosidic linkages (Czaja et al. 2007), which hold the cellulose polymer, resulting in the linear chain polymer having a large number of –OH groups. This strong structure can be propagated to molecules containing 1000–1500 β-d-gluclose monomeric units (Aravamudhan et al. 2014).

### Cellulose derivatives

**Fig. 4:**
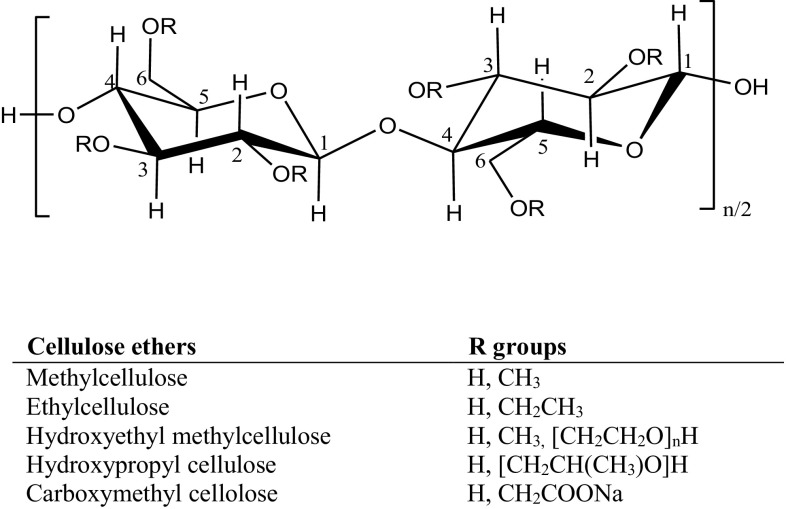
Chemical structure of ether derivatives

Most of the water-soluble cellulose derivatives are obtained by etherification of cellulose, where the active hydroxyl groups of cellulose react with organic species, such as methyl and ethyl units (Tosh 2015) (Fig. 4). Cellulose ether derivatives include methylcellulose (MC), ethylcellulose (EC), hydroxyethyl methylcellulose (HEMC), hydroxypropyl cellulose (HPC) and sodium carboxymethyl cellulose (CMCNa) (Marques-Marinho and Vianna-Soares 2013). The average number of etherified hydroxyl groups in a glucose unit determines the degree of substitution, which is controlled in such a way that cellulose derivatives can acquire the desired solubility and viscosity in water solutions (Mischnick and Momcilovic 2010).

**Fig. 5:**
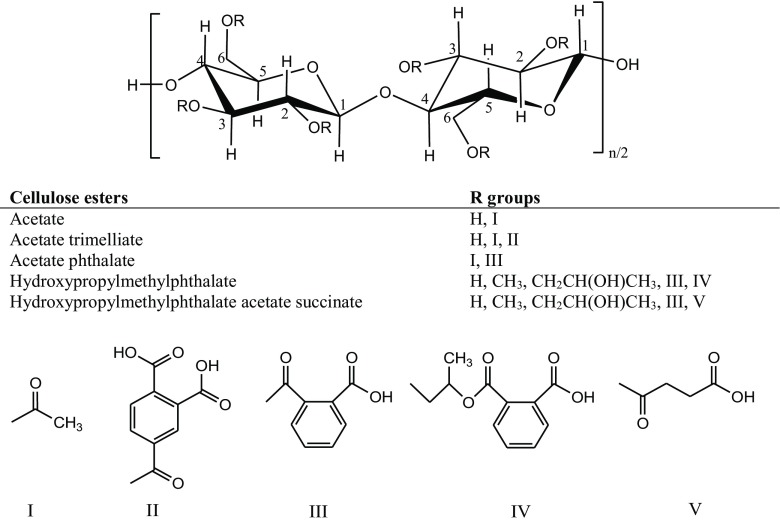
Chemical structure of cellulose ester derivatives

Cellulose ester derivatives are also used in the pharmaceutical domain for their unique functional properties. Here, these polymers are formed by esterification of cellulosic hydroxyl and various organic acids in the presence of a strong acid as a catalyst (Fig. 5); for example, cellulose acetate, acetate trimellitate, acetate phthalate, hydroxypropylmethylphthalate and hydroxypropylmethylphthalate acetate succinate (Marques-Marinho and Vianna-Soares 2013).

### Cellulose-based composite hydrogels

Cellulose-based composite hydrogels are made by blending natural biodegradable polymers or synthetic polymers with cellulose or its derivatives such as chitin, chitosan (Long and Luyen 1996; Mahmoudian and Ganji 2017) and starch (Faroongsarng and Sukonrat 2008) to achieve a new structural design and functional properties (Bajpai et al. 2008). For instance, phase-separated composite hydrogels have been prepared by blending cellulose and chitin in thiourea aqueous solution in the presence of H_2_SO_4_ as coagulant (Zhou et al. 2004). Cellulose powder and chitosan solution are mixed together to form cellulose/chitosan hydrogel beads, which are crosslinked with ethylene glycol diglycidyl ether to make them considerably denser and chemically stable for Cu adsorption applications (Li and Bai 2005). In addition, cellulose/starch composite gel can be prepared by keeping their homogeneous mixture for several days (Kadokawa et al. 2009). CB composite hydrogels can also be prepared from a synthetic polymer like polyvinyl alcohol (PVA).

CB composite hydrogels with PVA are cross-linked using several methods, including chemical agents, electron beam or physically thermal cycling (Ivanov et al. 2007). Another special type of water-soluble hydrogel called polyelectrolyte complexes can be constructed by introducing other positively charged polyelectrolytes in the presence of CMC (Papadakis and Tsitsilianis 2017). Chitosan and CMC solution are blended to form an amphoteric hydrogel membrane followed by crosslinking with glutaraldehyde. This hydrogel is flexible and can bend toward either anode or cathode, depending on the pH of the solution (Sperling 1994). CB composite hydrogels can be fabricated by interpenetrating polymer networks (IPNs). It is the combination of two or more polymers in the network that are synthesized in juxtaposition. Generally, two types of IPNs including sequential IPN and semi-IPN are employed for the fabrication of such hydrogels. For sequential IPN, cellulose is used as the primary network, and the secondary network is formed by polymerization in the presence of the cellulose network and in case of the semi-IPN hydrogel, the cellulose or its derivative is linear or branched in a crosslinked network (Nakayama et al. 2004).

### Cellulose-inorganic hybrid hydrogels

Cellulose-inorganic hybrid hydrogels are designed by incorporating inorganic contents into CB hydrogel system to improve functional performance. Recently, these types of hydrogels have become the focal point for their promising applications in electric, optical, magnetic and biological fields (Chang and Zhang 2011; Nie et al. 2005). Kumar et al.(2017) prepared cellulose-inorganic hydride hydrogel from cellulose nanocrystals by reinforcing with polyacrylamide/sodium alginate/silica glass, which showed improved mechanical properties in terms of good compressive stiffness together with high porosity, interconnected pore structure, high degradation stability, thermal stability, good cell adhesion and proliferation making them suitable for bone tissue engineering applications. Besides, the biomineralized thermo-responsive injectable hydrogel was developed by block copolymerization of hyaluronic acid (HA) and poloxamer (obtained from poly(ethylene oxide)/poly(propylene oxide)/poly(ethylene oxide) (Huh et al. 2015).

### Principal properties of hydrogels

#### Swelling properties

**Fig. 6:**
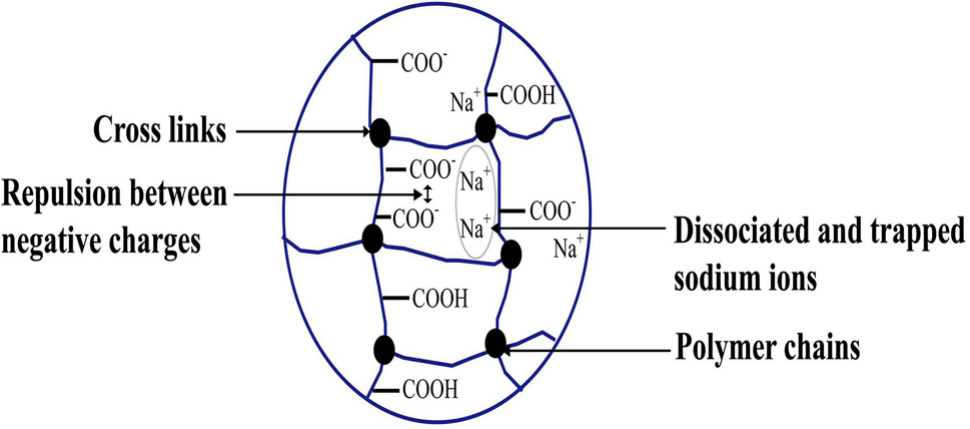
Swelling mechanism of hydrogels. Reproduced from Gooch (2011) with permission from the Springer Nature

The swelling mechanism of hydrogels explains the swelling behavior and underlying reasons for retaining three-dimensional structure in solvent system. Hydrogels containing hydrophilic group(s) and the dissociated sodium carboxylate group increase osmotic pressure in the gel that leads to swelling of hydrogels. Repulsion between negative charges positively influences swelling by expanding polymer coils. On the other hand, crosslinked polymers in hydrogels do not let the solvent or water dissolve hydrogels and thus prevent infinite swelling as in Fig. 6 (Gooch 2011).

The factors on which swelling property and the degree of swelling depend on include network density, solvent nature, polymer solvent interaction parameter (Jin and Dijkstra 2010). Water or solvent appears as a plasticizer in a hydrophilic polymer network system. Swelling properties of hydrogels are elucidated by Flory–Rehner theory, equilibrium swelling theory, using Gibbs free energy as follows (Koetting et al. 2015).

1 $$\Delta G_{\text{total}} = \, \Delta G_{\text{mix}} + \, \Delta G_{\text{el}}$$

where, Δ*G*_total_ is the total free energy of the polymer network, Δ*G*_mix_ is the free energy contributions due to enthalpy of mixing, and Δ*G*_el_ is the free energy contribution due to the elastic retractile forces in the network. Initially, Δ*G*_mix_ has high negative magnitude (Δ*G*_mix_ ≪ 0), but Δ*G*_el_ is positive with a lower magnitude than Δ*G*_mix_ (Δ*G*_el_ > 0). Therefore, their resultant effect is negative (Δ*G*_mix_ + Δ*G*_el_ < 0). At this stage, swelling starts and diffusion of solvent occurs into the network. During swelling, both are increased until their magnitude becomes zero, $\left| {\Delta G_{\text{mix}} } \right|\; = \;\left| {\Delta G_{\text{el}} } \right|$, so that the total free energy become zero, $\Delta G_{\text{total}} \, = \,\Delta G_{\text{mix}} \, + \,\Delta G_{\text{el}} \, = \,0$. Under this circumstance, there is no driving force for swelling, meaning that swelling stops and thus equilibrium of swelling is achieved (Yang 2012).

### Mechanical properties

To explain mechanical behaviors of hydrogels, rubber elastic theory is regarded as the most efficient approach, because it associates important bulk property of hydrogels: rubbery modulus scales, temperature, and crosslinking density (De Gennes 1979; Flory and Rehner 1943; James and Guth 1943; Wall 1942). Hydrogels show elastic property like rubber; however, original rubber theory cannot properly explain the nature of hydrogels in aqueous medium and demands some modifications (Chai et al. 2017). Later, Peppas et al. (2000) developed an Eq. (2) being known as rubber elastic theory is given below, which may describe the structure of hydrogels in solvent.

2 $$\tau = \frac{{\rho {\text{RT}}}}{\text{Mc}}\;\left( {1 - \frac{{2{\text{Mc}}}}{\text{Mn}}} \right) \;\left( {\alpha - \frac{1}{{\alpha^{2} }}} \right)\;\sqrt[3]{{\left( {\frac{{\upsilon_{{2,{\text{s}}}} }}{{\upsilon_{{2,{\text{r}}}} }}} \right)}}$$

Here, *τ* is the applied stress to the polymer as a function of elongation, *ρ* is the polymer density, *R* is the universal gas constant, *T* is the absolute temperature, and *M*_c_ and *M*_n_ are the average molecular weights between and without crosslinks, respectively, *α* is known as the extension ratio, and *υ*_2,s_ and *υ*_2,r_ are the polymer volume fraction in the swollen and relaxed states (Koetting et al. 2015).

### Applications of cellulose-based hydrogels

#### Biomedical applications

Hydrogels are one of the most prominent classes of polymers convenient to be employed in biomedical fields. Hydrogels possess diverse properties with biocompatibility and resemblance to living tissue that allows ample opportunities for biomedical applications, particularly in drug delivery and release systems, wound dressings as well as tissue engineering (Caló and Khutoryanskiy 2015).

#### Drug delivery

Drug release system using hydrogels function by the delivery of drugs at desired locations (tissue, cells) in response to environmental stimuli such as light, temperature, pH, chemicalactions,electric and magnetic fields. The polymeric aggregation due to swelling and shrinking property of hydrogels causes the change in transmittance, hydrodynamic radius, and stimuli-induced intermolecular and intramolecular hydrogen bonding (Qiu and Hu 2013). As a result, the drugs loaded in the assembly could be released, while disassembling occurs followed by swelling to loosen the hydrogel structures because of the actions of proximate stimuli (Qiu and Hu 2013). Moreover, swelling also affects the response rate, since it has a significant effect on the composition of hydrogels (Caykara et al. 2006). Stimulus–response rate is promoted and retarded due to swelling and shrinking, respectively. Amazingly, the inclusion of cellulose and its derivatives at various reaction levels leads to structural and morphological changes in hydrogel system in terms of enhancement of pore sizes due to repulsive forces of intra carboxyl groups directing to a large swelling ratio (Chen et al. 2010; Ciolacu et al. 2012). Thus, cellulose incorporation in hydrogels makes them an appropriate candidate for a manageable drug release. Also, the beauty of CB hydrogels is their biocompatibility, which has been verified by viability test (Tan et al. 2011; Xu et al. 2010).

There are numerous research articles along with critical reviews (Caló and Khutoryanskiy 2015; De las Heras Alarcón et al. 2005; Deligkaris et al. 2010; Elbert 2011; Gaharwar et al. 2014; Hoffman 2001; Hoffman 2012; Hoffman 2013; Kashyap et al. 2005; Klouda and Mikos 2008; Li et al. 2012; Mano 2008; Peppas 1987; Satarkar et al. 2010; Seliktar 2012; Tomatsu et al. 2011; Van der Linden et al. 2003; Ward and Georgiou 2011) and books (Andrade 1976; Gerlach and Arndt 2009; Ratner and Hoffman 1976) on the biomedical applications of hydrogels with an special emphasis on controlled drug delivery. A recently published review article piles up studies dedicated solely to drug delivery perspective of biodegradable hydrogels based on chitosan, poly(lactic-*co*-glycolic acid) and bacterial cellulose (Villalba-Rodríguez et al. 2017). Temperature, pH and redox-sensitive hydrogels are mostly useful for drug delivery because the body’s biological mechanisms are mostly connected with these stimuli. For example, the development of pH-responsive carboxymethyl cellulose (CMC) based hydrogel membrane crosslinked with acrylateis initiated for drug release and wound dressing applications (Pal et al. 2006). Thermo-sensitive volumetric nature of hydroxypropyl cellulose (HPC) and poly(*N*-isopropylacrylamide) (PNIPA) hydrogels are identified for biomedical applications (Marsano et al. 2004). Thiolated hydroxypropyl cellulose as redox and temperature sensitive hydrogel for controlled drug delivery and smart materials has been proposed by Tan et al. (2011). Oral drug release application using pH/temperature sensitive CB hydrogel from phyllostachys heterocycla is anticipated by Zhou et al. (2014). In addition, Bai et al. (2012) designed pH and thermo-responsive hydrogel from emulsion polymerization of poly (l-glutamic acid-2-hydroxylethyl methacrylate) (PGH) and hydroxypropyl cellulose-acrylic acid (HPC-AA) for a managed delivery of oral insulin.

**Fig. 7:**
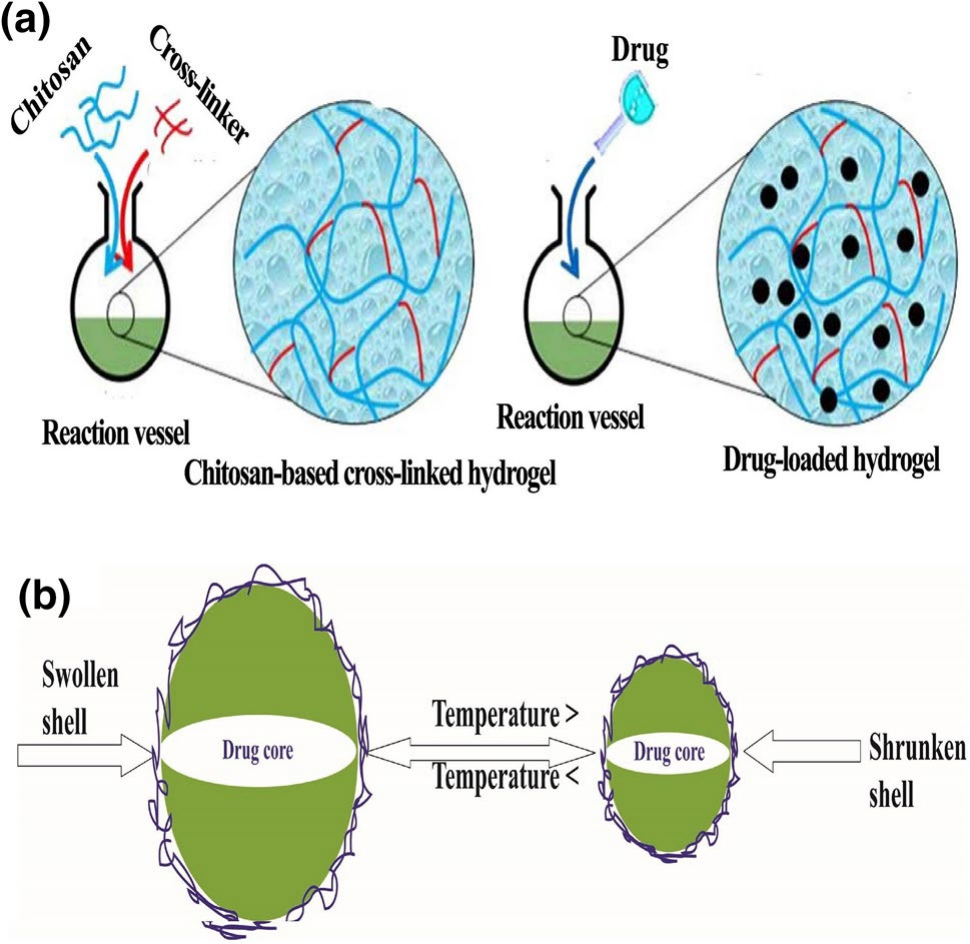
Schematic diagram of **a** loading of drug in the chitosan-based hydrogel structure during crosslinking reproduced from Villalba-Rodríguez et al. (2017) and **b** a temperature responsive hydrogels as controlled drug carriers

Controlled release of other proteins including dextran, ketoprofen, bovine serum albumin (BSA) and alaptide have been reported by some studies through the development of IPN and semi-IPN HPC-PAA, polyacrylamide-grafted-xanthan (PAAm-g-XG) and other CB hydrogels (methylcellulose, hydroxyethyl cellulose and hydroxypropyl cellulose), respectively (Chen and Fan 2008; Kulkarni and Sa 2008; Sklenář et al. 2013; Xu et al. 2010). Dutta et al. (2016) have prepared two types of triple (pH, temperature and redox) stimuli responsive hydrogels from carboxymethyl cellulose (CMC) and poly(*N*-isopropylacrylamide), where one is prepared by copolymerization (copolymerizing *N*-isopropylacrylamide) and the other one, methacrylated carboxymethyl cellulose, is by polymerizing NIPA in the presence of CMC. To make them redox responsive, two types of redox responsive crosslinkers (*N*,*N′*-methylenebisacrylamide (BIS) and *N*,*N*′-bis (acryloyl) cystamine (BAC)) are used. BAC crosslinked polymer is found to have higher swelling property than BIS crosslinked one, and lysozyme loaded in the hydrogels is released manageably through controlling temperature, pH and glutathione as a reducing agent. Besides, the release of BSAis marked by superabsorbent CB hydrogels (CMC and cellulose in the NaOH/urea aqueous system crosslinked with epichlorohydrin (ECH)) through shrinking and swelling mechanism (Chang et al. 2010). The drug loading procedure in the presence of crosslinking agent is presented in Fig. 7a, and drug control release mechanism of a temperature responsive hydrogels that swells and releases drug based on a set temperature and vice versa (Fig. 7b).

Target drug release is another crucial aspect of drug delivery for a precise and desired demonstration. Shen et al. (Shen et al. 2016) summarized drug release applications of stimuli-responsive CB hydrogels (cellulose/CMC and cellulose/lignin crosslinked with ECH, and cellulose/cellulose nanowhisker (CNW)) that are sensitive to chemical species (Ciolacu et al. 2012; Wang and Chen 2011; Xu et al. 2010), pH (chitosan/alginate crosslinked with Ca2 +/tripolyphosphate (TPP), chitosan/pectin crosslinked with Ca^2+^ and SO_4_^2−^, cellulose/PAAm, or PAAc crosslinked with *N*,*N′*-methylenebisacrylamide (MBAAm)) (Anal and Stevens 2005; Bigucci et al. 2008; Chang and Lin 2000; Xu et al. 2007), magnetic field (cellulose/Fe_3_O_4_glutaraldehyde (GA)), injectable (chitosan/CMC, chitosan/collagen, chitosan/polyethylene glycol (PEG)) (Bhattarai et al. 2005; Chen and Fan 2008; Chiu and Radisic 2011), temperature (cellulose/PNIPAAm crosslinked with MBAAm, cellulose/poly(*N*-vinylcaprolactam) (PNVCL) crosslinked with MBAAm, chitosan/PNIPAAm crosslinked with MBAAm and GA, chitosan/PNVCL) (Alvarez-Lorenzo et al. 2005; Montes et al. 2015; Sanna et al. 2013; Wang et al. 2013). Among these hydrogels, CB hydrogels facilitate a desired delivery of drugs at around neutral medium, whereas chitosan-based hydrogelsare at a lower pH medium. The base whether pure cellulose or chitosan-based hydrogel to be used is defined by some criteria such as pH, temperature and chemical species. Because, for example, pH of different organs of human body are different and ranges from 1 to 7.5 (e.g., saliva 5–6, stomach 1–3, intestine 6.6–7.5, and colon 6.4–7.0) (Bhattarai et al. 2010).

On the other hand, Feketeet al. (2017) mentioned that carboxymethyl cellulose/starch superabsorbent hydrogels prepared by gamma-irradiation works better than pure CB hydrogel in the context of a higher water uptake at a higher electrolyte concentration. Chitosan/pectin hydrogels are responsible for colon-specific delivery for their muco-adhesion characteristics and enzyme-dependent degradation. Again, pH sensitive hydrogels can be assigned as prospective oral drug carriers for intestine or colon-specific delivery. They offer target drug carriers without employing invasive surgeries leading to extending release time and encapsulating drugs. Ahmadi et al. (2015) mentioned multi-dimensional drug delivery system of chitosan-based hydrogels for oral, ocular and nasal drug delivery. For dermal and transdermal drug delivery systems, use of cellulose-derivatives-based hydrogels have been elucidated by Vlaia et al. (2016). Target protein delivery using Fe_3_O_4_ nanoparticles in the regenerated cellulose microspheres is developed by establishing magnetic-induced transference (Luo et al. 2009).

Cellulose-based hydrogels hold a great promise for technological advances in the early detection and treatment of diseases. Nanoparticles like lipid-based micelles and liposomes, polymeric micelles and dendrimers, carbon nanomaterials, and inorganic and metallic nanoparticles are being used for numerous applications such as site-specific drug delivery, biosensors for in vitro and in vivo diagnostics and contrast agents for medical imaging (Roman 2017). Cellulose derivatives are also used to meet different requirements such as fillers, solubility and bioavailability enhancers of active pharmaceutical ingredients, to ease manufacturing of dosage forms or to achieve a certain release profile from formulations (Grace Okoh 2017).

### Wound healing

Wound healing is a natural restorative response that can be used to fix the damaged tissues. Healing is the interaction of a complex cascade of cellular events that generate resurfacing, reconstitution and restoration of the tensile strength of injured skin (Patrick et al. 2016). It is also a complex and dynamic process of replacing devitalized and missing cellular structures and tissue layer (Michael Mercandetti 2017). CB hydrogels and their derivatives are commercially available as wound dressing products in different forms such as fibers, membranes and sponges (Jayakumar et al. 2011; Nagahama et al. 2008). The main reason is that deacetylated chitin or chitosan is a hemostat and polycationic in nature, so it possesses a natural antimicrobial property (McCarthy et al. 2005). Usually, cellulose itself has no antimicrobial activity to prevent wound infection and thus ZnO or Ag nanoparticles (NPs) are impregnated into the cellulose gel system to confer antimicrobial properties (Katepetch et al. 2013; Li et al. 2011; Maneerung et al. 2008). However, chitin and chitosan possess hemostatic, antimicrobial and permeable properties, therefore, dressing materials made of those polymers are non-irritating and non-allergic (James and Guth 1943; Khan et al. 2000; Mi et al. 2001). The porous and the interconnected 3D structures of hydrogels aid the growth of the cells, also the easy modification of the polymers allows them to act as strong and multipurpose carriers for drug conveyance. Nowadays, novel injectable hydrogels can substitute risky surgeries (Shamma et al. 2017).

Chitosan derivatives exhibit an enhanced bacteriostatic activity compared to pure chitosan and show few interesting properties in self-healing applications (Prabaharan and Mano 2006). In addition, recent applications are associated with various types of ‘intelligent’ self-healing and anticorrosion coatings that use the stimuli-responsive behavior of polymer-based nanoparticles (Motornov et al. 2010). Yang and Lin (2004) developed temperature-responsive wound dressing materials from polypropylene nonwoven fabric (PP) and acrylic acid (AA) by γ-irradiation-induced method to achieve PP-*g*-AA and modified by *N*-isopropylacrylamide (NIPAAm) through graft-copolymerization along with ultraviolet photografting to achieve PP-*g*-AA-*g*-NIPAAm bigraft nonwoven fabric. Then chitosan was impregnated onto (PP)-*g*-AA-*g*-PNIPAAm bigraft nonwoven fabric material. Their research finds the evolution of relative wicking time and the antibacterial assessment on the dressing materials. The relative wicking time was found to decrease for the PP-*g*-AA-*g*-PNIPAAm-chitosan. Studies on the transport of bacterial and water vapor transmission rates (WVTR), the permeance and the permeability through the modified nonwoven fabric, have also been conducted. As pure PP is porous, it shows the highest values of WVTR and permeance. After some changes, the PP-*g*-AA-*g*-PNIPAAm forms a dense cross-section, so the permeance and permeability values of WVTRdrop sharply. Relative wicking time of PP-*g*-AA-*g*-PNIPAAm increases with increasing the temperature and then levels off for temperatures above 328 °C due to the thermo-sensitive behavior of the PNIPAAm. Since chitosan is a cationic polysaccharide and the bacterial cell surfaces are negatively charged, the adsorption of the negatively charged cell surfaces onto the nonwoven fabric increases with increasing the charge density on the chitosan containing PP-*g*-AA, resulting in a decrease in the viable cell number. The modified nonwoven fabric with chitosan can be considered for wound dressing, as the values of WVTR, permeance and permeability of the PP-*g*-AA-*g*-PNIPAAm-chitosan are comparable to the commercial products (Yang and Lin 2004).

### Tissue engineering

Tissue engineering refers to the most recent applications of CB hydrogels, in which they work as scaffolds to mimic cellular functions of extracellular matrixes and to engender new tissues (Radhakrishnan et al. 2015). For example, scaffolds provide proper conditions (space and nutrients) for a desired new tissue generation and potentially regulate the structure and function of the engineered tissue in situ or in vitro (Lee and Mooney 2001; Shen et al. 2016). In addition, hydrogel scaffolds are at near clinical uses in engineering many tissues in the body including cartilages, bones, muscles, skins, fats, arteries, ligaments, tendons, livers, bladders and neurons (Marler et al. 1998). Apart from clinical usage, hydrogel can be used as surface modification in tissue growth. Synthetic materials working as biomedical implants/supports for tissue growth require surfaces that either resist attachment of certain cells while binding others or being capable of binding a biological moiety under some conditions (Okano et al. 1995). This surface modification method can be a good option to control and modulate cellular interaction through preventing the adhesion of unwanted cells and promoting interaction between bone and skin with implant biomaterials. Applications define whether the surface of a biomaterial should promote cell adhesion or avoid the attachment of specific proteins and cells (Tallawi et al. 2015). Okano and co-workers (Harimoto et al. 2003; Shimizu et al. 2003; Shiroyanagi et al. 2003; Yamato et al. 2002; Yamato et al. 2001) have extensively used thermo-responsive PNIPAAm-based polymers as surface mediators of biopolymer and cell attachment. Tissue engineering also focuses on the replacement of damaged or infected tissues or organs that helps the body regenerate new functional tissues (Prabaharan and Mano 2006). According to Prabaharan et al. (2006), this is usually achieved through constructs containing living cells, a three-dimensional porous matrix or scaffold, and bioactive molecules. The formation should accelerate cell attachment, proliferation and differentiation. The scaffold materials should have an extensive set of properties such as biocompatibility, biodegradability, mechanical strength, porosity, potential of entrapment and release of pharmaceutically active agents.

Thermo-responsive hydrogels (i.e., chitosan-*g*-PNIPAAm) are good solution to avoid the challenges in mesenchymal stem cells (MSCs) culture. An et al. (2001) discussed various challenges facing culture of MSCs. The most visible drawback arises upon building MSC from the cell culture carrier because of high adhesive nature of MSC that involves application of harsh conditions. Therefore, a further study is required regarding cell culture carrier materials and gentle harvesting of the cultured cells. One method is the use of thermo-responsive hydrogels as cell culture carrier. By using thermo-responsive hydrogels, the cultured cells can be reformed by simply decreasing the temperatures below their lower critical solution temperature. Chitosan-*g*-PNIPAAm has decent characteristics for cellular attachment, proliferation and viability as well as differentiation into chondrocytes (An et al. 2001; Chenite et al. 2000). Due to these properties, an injectable gel material based on chitosan/PNIPAAm has been developed for chondrogenic differentiation of human MSCs and evaluation of cartilage formation in vivo after injecting a cell-thermo-sensitive gel complex (Gil and Hudson 2004). A further experiment on animal is performed to assess the cartilage formation in the submucosal layer of the bladder of rabbits. The thermo-sensitive gels system is opposite to in vivo applications, because its lower critical solution temperature (LCST) is 3200 °C. As a result, this new combination (chondrogenic differentiated cells from MSCs with a thermo-sensitive polymer) is suggested to use as an injectable cell-polymer complex. Chondrogenic differentiation is found to be induced from the MSCs both in vitro and in vivo applications. The gel is recommended for obtaining an easy method for treating vesicoureteral reflux via an endoscopic single injection technique (without a dual injection system) (Cho et al. 2004).

### Smart materials

CB hydrogels have been gaining popularity in fabrication of smart devices because of their overall biocompatibility, high storage capacity for cells and small molecules and low interfacial tension at the gel-aqueous solution interface (Tokarev and Minko 2009). Smart device like biochemical sensor translates chemical coding that includes the concentration of a specific sample component to a total composition analysis. Chemical sensors usually have two basic components connected in series: a chemical (molecular) recognition system (receptor) and a physio-chemical transducer. Biosensors are chemical sensors in which the recognition system utilizes a biochemical mechanism (Thévenot et al. 2001). Smart materials based on cellulose has unique properties such as strong mechanical strength, and biocompatibility, thus studies on ‘smart’ materials based on cellulose have been abuzz during the last decade (Qiu and Hu 2013). Cellulose particles, their derivatives and composites are abundant in nature with their various eco-friendly applications. They possess cellulosic properties and are used in electro-responsive electro-rheological (ER) suspensions (Choi et al. 2017). Cellulose composite particles with carbon nanotube have extensive applications in conductors, wearable electronics, structure health monitoring, smart textiles, detection of liquids (such as water) leakages, strain sensor, thermistor, humidity or vapor sensors, strain sensor, biosensor, chemical sensors (gases or vapors), batteries, energy storage and supercapacitors (Qi 2017).

Stimuli-responsive polymer systems accelerate an efficient transduction mechanism that helps to prepare them suitable for use in sensor applications. Hydrogel film can be used as a highly sensitive pH-responsive nano-sensor with short response times (Tokareva et al. 2004). According to Guenther and Gerlach (2009), a quick expanding field of on-line process monitoring for biotechnology, food industry, the pharmaceutical industry, process chemistry, environmental measuring technology, water treatment and sewage processing requires the development of new microfabricated reliable chemical biosensors that can process analytic information in a faster, simpler and economical manner. Biosensors being coated with functionalized hydrogels can detect transmit and record the information regarding the concentration change, the presence of specific functional groups or data by producing a signal. This signal is proportional to the concentration of the target analyte containing organic and inorganic contaminants (Guenther and Gerlach 2009). The sensitivity of hydrogels depends on a large number of physical factors such as temperature (Kuckling et al. 2003; Richter et al. 2004), electrical voltage (Richter et al. 2003), pH (Kuckling et al. 2003), concentration of organic compounds in water (Kumar et al. 2006) and salt concentration (Liu et al. 2003). Stimuli-responsive hydrogels are capable of reversibly converting chemical energy into mechanical energy that makes them very useful as a sensitive material for sensor applications. The following principles have been explored by Guenther and Gerlac (2009) for measuring the environmental parameters used in sensors based on the swelling behavior of hydrogels including modification of the holographic diffraction with respect to wavelength in optical Bragg-grating sensors, shifts of the resonance frequency of a quartz crystal microbalance in microgravimetric sensors, bending of micromechanical bilayer cantilevers and deflection of a membrane or bending plate1 in capacitor/inductor micro-machined resonator and in piezo-resistive pressure sensors.

The swelling properties of pH-sensitive hydrogels vary based on functional acidic or basic groups in the polymer backbone. The density of ions in the hydrogel is higher than that in the surrounding solution because of dissociation of these groups and the influx of counterions. This leads to a difference in osmotic pressure and solution flux into the hydrogel as well as in swelling properties. The interaction and repulsion of charges along with the polymer chain also cause to an increased swelling. Apart from sensitivity of pH and chemical composition, signal reproducibility and long-term stability are the most crucial aspects for a successful sensor implementation. The excellent long-term stability of the sensor is governed by the stability of the hydrogels. The polymer film preparation and measurement conditions have been determined, which are necessary for a high signal reproducibility and high long-term stable sensor sensitivity. In order to provide the required high signal reproducibility of the sensors, two procedures were employed to run the process: (i) an initial gel conditioning procedure consisting of a gel swelling in de-ionized water and a subsequent deswelling/swelling cycling and (ii) a regenerating procedure prior to every measurement in order to achieve a certain reference sensor signal. In deeper sense, stimuli-responsive polymer-grafted membranes can change their pore sizes through swelling and shrinking in response to external stimuli. These regulated membranes are designed to separate membranes and sensors (Qiu and Hu 2013). Recently, Richter (2010) has found that CB hydrogels follow this design principle in microfluidics and special imaging systems. Automatic microfluidic systems based on the sensor-actuator properties of hydrogels offer functionalities, which have not yet been realized with other systems or actuators. The author also shows the basic principles applied to an electronic control for hydrogel actuators and also to the basic components for microfluidics: microvalve, micropump and hydrodynamic transistors.

### Healthcare and hygienic products

History reveals that the application of hydrogels as SAPs in the realm of personal healthcare and hygienic feminine products is quite phenomenal. The insoluble hydrogels by nature have a high liquid absorption capacity, which is one of the crucial requirements of SAP that can absorb water 1000 times of their initial dry weight. Their exceptional swelling property also assists to enlarge the pore size so that a large amount of secreted liquids like blood, urine can be engrossed in the hydrogel structure (Zohuriaan-Mehr and Kabiri 2008). Subsequently, hydrogels are widely applied for designing disposable diapers, napkins, adult urinary incontinence products and personal safety products (Klinpituksa and Kosaiyakanon 2017). Besides, SAPs showed a better performance than multilayered diapers and synthetic over pants providing protection against diaper rash, spreading germs, leakage of diapers for minimizing fecal contamination, gastrointestinal illnesses (Adalat et al. 2007; Holaday et al. 1995).

**Fig. 8:**
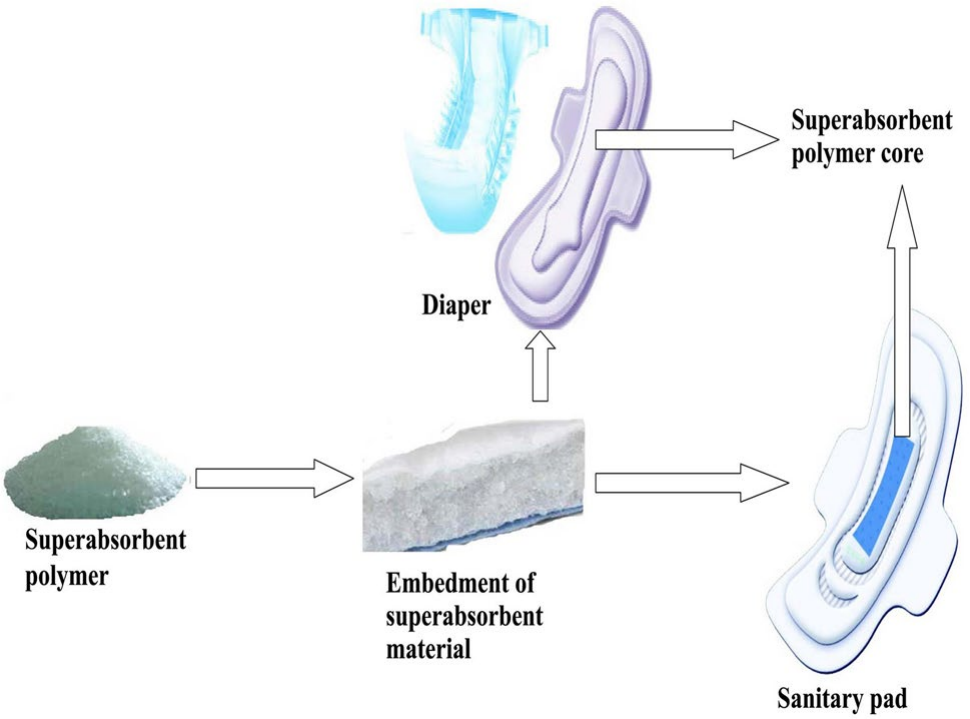
Super absorbent polymer embedment in diaper/napkin system

Despite the use SAP for the personal disposable purpose, it is first patented in 1972 (Atkins et al. 1972; Harmon 1972), the commercial use of SAP as sanitary napkins came into existence in 1982, which was introduced by Unicharm Japan. In their invention, they embed superabsorbent polymer in layer by layer form between the top and bottom sheet of the diaper/napkin system (Fig. 8). This revolutionary innovation has earned tremendous popularity throughout the globe due to its extraordinary liquid retention power with reduced diaper rash and leakage risk (Sannino et al. 2009). Nowadays, more than 90% of the diapers, napkins and other hygiene products are made of synthetic polymers mostly based on acrylate such as acrylic acid or acrylamide (Friedrich 2012). Some obvious drawbacks associated with synthetic SAHs include toxicity, non-biodegradability, expensiveness, environmental unfriendliness and huge waste load after disposal (Ma et al. 2015). Later, with the development of SAH from flax yarn waste overcomes the limitations of the traditional synthetic hydrogels (Liu et al. 2014). Besides, CB hydrogels such as CMC and hydroxyethyl cellulose (HEC) crosslinked with divinyl sulphone show improved swelling property and a higher water uptake percentage under centrifugal loads compared to their synthetic counter parts and thus turn out to be a suitable alternative tothe synthetic SAHs (Sannino et al. 2004).

Seki et al. (Seki et al. 2014) introduced CMC and HEC based hydrogels responsive to pH and salt. They assessed the water absorption capacity and tensile strength of the newly developed hydrogels at different pH and salt concentrations and showed that water absorption is higher at neutral pH (7.4) and becomes lower with decreasing charge of metal ion of salt. Besides, the incorporation of microporous structure in the hydrogels through phase inversion desiccation technique in the presence of acetone results in an enhancement of water retention and swelling kinetics due to capillary effects (Onofrei and Filimon 2016; Sannino et al. 2009). The development of biodegradable, renewable and recyclable CB hydrogels using non-toxic crosslinker such as, gamma irradiation crosslinking for the modernization of SAH in the domain of hygienic and personal healthcare products has been investigated in some contemporary research. Barleany et al. (2017) proposed that acrylic acid being neutralized with KOH and mixed with chitosan to form superabsorbent poly(acrylic acid)-graft-chitosan hydrogel by gamma irradiation technique can improve water absorption capacity. Cellulose-based SAP through the inverse suspension polymerization, using cellulose, acrylic acid and acrylamide as monomers having humidity resistant property, and a higher water uptake capacity was prepared by Guan et al. (2017). In addition, Peng et al. (2016) proposed a novel CB hydrogel prepared by chemical crosslinking of quaternized cellulose (QC) and native cellulose in NaOH/urea aqueous solution in order to overcome some inherent weaknesses including poor mechanical property, inferior biocompatibility as well as lack of antimicrobial activity associated with the existing SAHs employed in commercial disposable diapers.

### Agricultural use

In agriculture, proper irrigation and supply of nutrients are pressing needs for a favorable germination and upbringing of plants and crops (Ismail et al. 2013). Hydrogels of SAPs, alternatively SAHs, have been widely used during past five decades in various aspects of agricultural system including an enhancement of soil property (permeability, density, structure, texture, water evaporation, filtration rate) (El-Rehim et al. 2004), release and carry agrochemicals (Li et al. 2009b), assist in growing plant in both drought affected and arid areas (Woodhouse and Johnson 1991), stimulate sprouting (Knypl and Knypl 1993), rise nodulation between actinorhizal plant species (Kohls et al. 1999) as well as performing as soil additive (Lokhande and Varadarajan 1992). Moreover, SAHs can also be employed in cultivating artificial edible products (Zohuriaan-Mehr et al. 2010), ornamentation of plants (Abedi-Koupai and Asadkazemi 2006), developing properties of horticulture substrate (bark, perlite, peat, and their mixtures) (Martyn and Szot 2001), establishing biotechnical protection (Orzeszyna et al. 2006), promoting sand stabilization (Han et al. 2007; Jafarzadeh et al. 2005), motivating bacterial aggressiveness (Lee et al. 2008), control erosion, standing water and petroleum spills (Zohuriaan-Mehr et al. 2010). All these agricultural and horticultural applications of SAHs can be categorized under two broad segments namely; water reservoir and nutrient carrier.

**Fig. 9:**
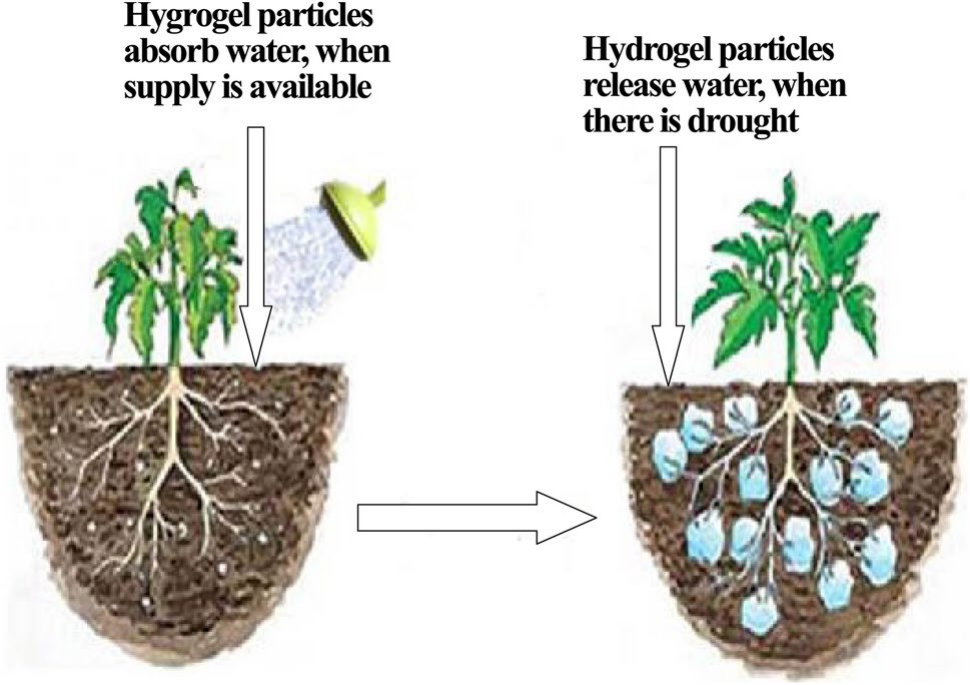
Change of soil porosity with the swelling of hydrogels

The soil is extensively arid in the drought-affected regions of the world such as Africa, South America, east of Asia, which poses a great threat to the plants/crops (Ma et al. 2015). SAHs have gained much popularity for the application in that regions through eliminations the soil aridity via moisturizing as well as improving water retaining capacity of the soil for an extended period of time (Guilherme et al. 2015). After mixing the soil with hydrogels, a huge amount of water is stored by swelling of hydrogels upon watering either naturally or by irrigation. This phenomenon enables soil particles to turn from glassy to rubbery state even under compressive force. On the other hand, releasing occurs very slowly through the diffusion-driven mechanism in the presence of humidity gradient developed between the outside and inside of the hydrogels (Ma et al. 2015). Therefore, a large amount of water is retained in the hydrogel structure that eventually releases slowly when required (Fig. 9). This process also decreases the frequency of irrigation and thus reduces a significant amount of water that could serve other purposes. At the same time, it also saves the reserved water in the hydrogel from evaporation and drainage. Another interesting thing worthy to be noted is due to swelling effect, hydrogel granules being almost similar in size to other particles in the dry state get bigger upon absorbing water. As a result, the compact hydrogel-soil interaction becomes loose that ultimately causes more oxygenation and growth of plant root by increasing soil porosity (Sannino et al. 2009).

Nowadays, essential and compatible nutrients are fabricated with hydrogels and applied altogether during cultivation. Loading stimulus-responsive hydrogels, functional services of hydrogels can be improved with respect to the controlled release of agrochemicals (drugs, herbicides or pesticides). These conglomerations mitigate environmental pollution due to agrochemicals and also ensure smooth and continuous supply of those chemicals to the plants, because they are already retained in the hydrogel structure (Ismail et al. 2013). As mentioned earlier, most of the SAHs being used today are synthetic hydrogels (mostly acrylate-based), making themselves as soil pollutant because of their non-biodegradable nature. CB hydrogels are thus of great interest in academic and industrial research due to their environmentally friendly nature in terms of biodegradability, renewability, non-toxicity, non-petrochemical property. Cellulose-based hydrogels from chitosan, CMC, starch and other polysaccharides have been used to optimize water reservoir (Cannazza et al. 2014; Demitri et al. 2013; Montesano et al. 2015; Sánchez-Orozco et al. 2017). The use of hydrogel from CMC is also found suitable for a controlled release of herbicide like acetochlor based on diffusion mechanism (Li et al. 2009a; Li et al. 2008). Cellulose from palm oil-free fruit bunch along with polyacrylic acid hydrogels is found to improve soil-retaining capacity and urea leaching loss rate of sandy soil (Laftah and Hashim 2013). Hydrogels made of polyacrylamide (PAAm), methylcellulose and calcic montmorillonite have been proved to impart controlled release of fertilizer through volatilization of ammonia (Bortolin et al. 2013). Furthermore, to highlight applications of hydrogels as nutrient carriers, different types of CB hydrogels for releasing nutrients, acids, fertilizer, herbicide, urea etc. are summarized in a study (Guilherme et al. 2015). Hydrogel being synthesized from CMC and polyvinyl pyrrolidone (PVP) crosslinked with gamma irradiation finds versatile agricultural applications ranging from urea release to good water retention capacity (Wang and Wang 2010).

### Applications in textile processing

**Fig. 10:**
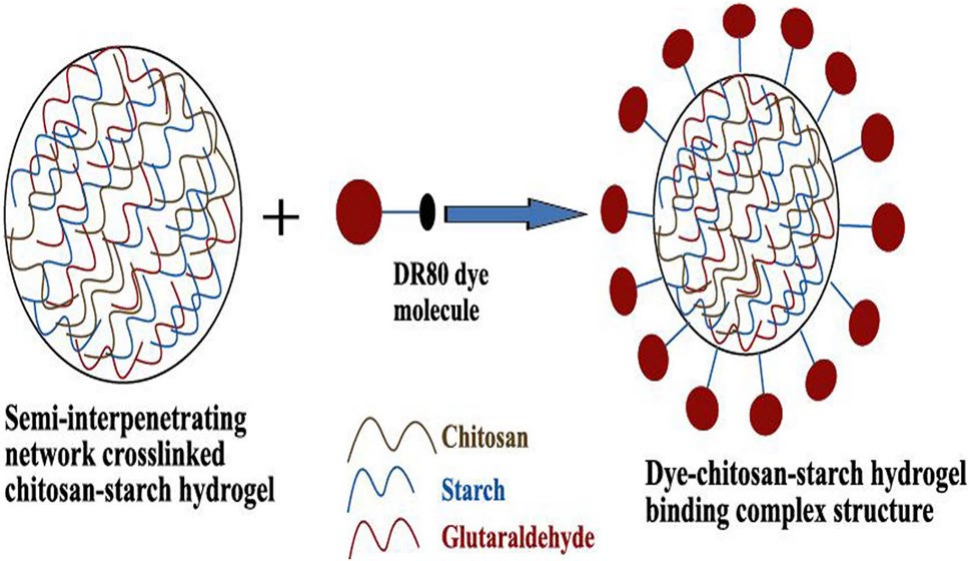
Adsorption mechanism of DR80 dye onto chitosan-starch hydrogel surface (reproduced from Ngwabebhoh et al. (2016) with permission from the Elsevier)

Cellulose hydrogel materials have garnered much attention for application in the field of textiles including the processing of textile materials and treatment of textile wastewater through adsorption. Some of their exceptional properties namely high absorbency, porous structure, rich functional groups and relatively lower crystallinity (Shen et al. 2016; Tang et al. 2011; Zhou et al. 2005) have touted CB hydrogels as a potential candidate for the application in textile wet processing (dyeing and printing) (Hu et al. 2012), development of heat and moisture management textiles (Hu et al. 2012) as well as disposable absorbent personal-care products with self-fitting capability (Topolkaraev and Soerens 2003). Besides, hybrid hydrogels with the incorporation of different particles can be employed to remove various water pollutants resulting from the textile wastewater such as metal ions (transition or radioactive) (Fu and Wang 2011; Li et al. 2005; Ngah and Fatinathan 2010; Ngah et al. 2004), dyes (cationic or anionic), (Azlan et al. 2009; Copello et al. 2011) and other ions (nitrogenous or phosphorous) (Chatterjee and Woo 2009; Zheng and Wang 2009). At present, many liquid and solid-phase extraction techniques have been widely used for the removal of toxic pollutants from wastewater including chemical precipitation, flocculation, flotation, coagulation, membrane filtration, ion exchange, adsorption and electrochemical treatments (Fu and Wang 2011; Shen et al. 2016). From a contemporary research, it has been revealed that cellulose hydrogels can also be employed efficiently to remove partially or fully dissolved pollutant substrates from wastewater by allowing them to penetrate deep into their structure and forming bonds with the pollutants through more reactive amine (–NH_2_) and/or hydroxyl (–OH) groups at their optimum pH values (Shen et al. 2016). The resulting bond formation occurs mostly due to three kinds of interactions that include complexation (or chelation) between the lone pair electrons of N and/or O and the metal ions (Li and Bai 2005; Tang et al. 2011; Tang et al. 2014; Zhou et al. 2005), crystallization of the metal ions with the complexed metals as nucleation sites (Fu and Wang 2011; Sklenář et al. 2013; Zhou et al. 2005) and electrostatic attraction (or ion exchange) between the protonated amino groups and various anions (Chatterjee et al. 2010; Chatterjee and Woo 2009). The superabsorbent chitosan-starch hydrogel shows excellent swelling ability in both water and DR80 (dye) solutions. Chitosan-starch hydrogel is efficient for DR80 removal under batch system. For successful sorption, dissociation of DR80 dye molecules and activation of chitosan-starch hydrogel functional groups induce electrostatic force of attraction (Ngwabebhoh et al. 2016). The binding of DR80 dye molecules and chitosan-starch hydrogel surface are bonded as shown in Fig. 10.

### Miscellaneous applications

SAHs based on cellulose also find themselves in a number of miscellaneous applications including construction purpose, electrical use, membrane and flocculant manufacturing, water swelling rubbers, artificial snow, packaging and re-freshening systems, enzyme and catalyst supports, fire-extinguishing gels, sludge/coal dewatering, body water retainers, stomach bulking agents and food preservatives (Zohuriaan-Mehr et al. 2010).

Constructional uses of SAHs mainly include preparation of cementation (Mignon et al. 2017b) and demolition of building structures (Ismail et al. 2013). Crack formation because of autogenous shrinkage, decreasing freeze/thawing and other constructional stresses are major concerns for building constructions. Synthetic SAHs have been successfully experimented to combat this problem through internal curing, self-sealing effect, autogenous healing (Mignon et al. 2017b). Kinetics and water release behavior of SAHs during cementations have been investigated recently by nuclear magnetic resonance (NMR) (Snoeck et al. 2017). However, this approach of using synthetic SAHs has also some innate limitations such as formation of macropores that adversely affects the concrete strength (Gupta et al. 2017; Mechtcherine et al. 2017; Mignon et al. 2017b; Schröfl et al. 2017). Some recent works (Mignon et al. 2017a; Mignon et al. 2016; Mignon et al. 2017c; Mignon et al. 2017d; Schröfl et al. 2017) have suggested use of cellulose-based SAHs in mitigating the strength problem due to excessive swelling of synthetic SAHs. In contrast, the application for demolition is based on the opposite principle, using highly absorbing hydrogels (Ismail et al. 2013). Kobayashi et al. (1990) have patented acrylonitrile-graft-starch hydrogel as demolishing agent that swells upon contacting water and thereby weakens the rigid structures through expansion.

CB stimulus hydrogels are utilized for designing electric cables as water blockers (Ismail et al. 2013) and electroactive devices (electrolytes, sensor, actuators, robot haptics) (Cunha et al. 2017). Hydrogels from homo-polymers, copolymers and/or mixtures of sodium and potassium polyacrylates, partial sodium salts of polypropenoic acid and starch-grafted sodium polyacrylates are used for wrapping cables in order to prevent water penetration (Ismail et al. 2013). Cunha et al. (2017) introduced electrolytes being comprised of microcrystalline cellulose (MCC) in aqueous LiOH/urea solvent, where CMC is blended at different levels. This recyclable/disposable, biodegradable, eco-friendly cellulose electrolyte hydrogel has some improved features such as high specific capacitances, transparency and flexibility making it suitable for engineering many electrical devices (sensors, transistors). Physical crosslinking of polyvinyl alcohol and cellulose generates cellulose electroactive hydrogel for actuator applications (Jayaramudu et al. 2017). Besides, cellulose and its derivative (chitin/chitosan/PVA) hydrogel membranes containing ILs, H_2_SO_4_, NH_4_NO_3_ and ethylene carbonate are used for capacitor/battery (Shen et al. 2016).

Cellulose-based hydrogel system is also used as electrolyte membranes and flocculants. Hydrogel from chitosan crosslinked with polyacrylic acid has been used in the fuel cell as a membrane for facilitating ion exchange mechanism (Smitha et al. 2004). Navarra et al. (2015) prepared CB hydrogel crosslinked with epichlorohydrin for gel electrolyte membrane applications. Hydrogels are applied to ease water purification process; for example, hydrogel prepared from guar gum has been proposed for water separation from wastewater (oil–water) by coating stainless steel mesh to render an excellent underwater oleophobicity and super-low oil sticking. Flocculation is the phenomenon of precipitation in the form of small lumps due to chemical interactions between soil particles and others such as salt water depending on settling time, temperature, number of polymers and pH of the solution. Psyllium based hydrogels (methacrylic acid, polymethyl methacrylate grafted psyllium) can act as cheap and green flocculants. Cationized CMC treated with *N*-(3-chloro-2-hydroxypropyl) trimethylammonium chloride (Hebeish et al. 2010) and hydroxypropyl methylcellulose grafted with polyacrylamide (Das et al. 2013) have been also found as outstanding flocculants in the light of the factors affecting flocculation efficacy.

Hydrogels by their nature exhibit crosslinking properties as explained by rubber elastic theory (Oyen 2014). Water swelling rubbers are like typical hydrogels used for construction applications as water blockers of underwater tunnels and subways so that water cannot pass through the structure in case of any leakage (Zohuriaan-Mehr et al. 2010). Hydrogel from natural rubbers comprising poly(ethyleneoxide) and glycidyl methacrylate (GMA) as a reactive coupling agent can be used as water swelling rubbers. Yang et al. (2013) prepared rubbery hydrogel by free radical polymerization of cellulose nanocrystals and polyacrylamide (without chemical cross-link) and analyzed network structure and mechanical properties that affirm that the prepared hydrogel can be applied in environmentally benign applications.

In addition, the materials used for packaging are mostly of synthetic nature and non-biodegradable. In this regard, CB hydrogels could offer a green solution to the packaging industry with enhanced mechanical strength. Roy et al. (2012) prepared hydrogel film from polyvinylpyrrolidone and CMC by solution casting method. The resulting new hydrogel film showed a better mechanical strength, biodegradability and ability to absorb moisture from fruits and vegetables. To augment hydrogel packaging system in terms of preserving food products, some successful efforts have been made by incorporating antimicrobial property that cannot only keep food safe and dry but also protect them from the attack of bacteria like *Escherichia coli* (*E. coli*). Strong antimicrobial hydrogel package could be made by graft copolymerization of ceric ammonium nitrate and CB filter paper in which silver nanoparticles are being embedded as an antibacterial agent (Tankhiwale and Bajpai 2009). Other notable applications of cellulose-based hydrogels in the various fields are also summarized in Table 2.

**Table 2:** Applications of CB hydrogels

Application fields	Hydrogels used	For	References
Enzyme and catalyst support	Bacterial cellulose (BC)-chitosan composite hydrogel	Enzyme supports for immobililization lipase	Kim et al. (2017)
	Cellulose-*g*-poly(acrylic acid)/poly(vinyl alcohol) semi-IPN hydrogel	Catalyst to reduce 4-nitrophenol (4-NP)	Ding et al. (2017)
	Chitosan hydrogel	Organocatalyst for aldol and Knoevenagel reactions	Reddy et al. (2006)
Fire-extinguishing gels	Corn straw acrylamide-2-methylpropanesulfonic acid and acrylic acid	Fire-extinguishing and preventing re-ignition	Cheng et al. (2017)
	Fire-Resistant Hydrogel-Fabric	Fire-resistant blankets or apparel	Illeperuma et al. (2016)
	Chitosan based hydrogel	Prevents coal spontaneous combustion	Hu et al. (2015)
Body water retainers	CMC and HEC	Water removal during edemas treatment	Esposito et al. (2005), Sannino et al. (2000), Sannino et al. (2003)
Stomach bulking agent	CMC and HEC	Bulking agent in dietary	Sannino et al. (2005)
	CB hydrogel	Bulking agent for rat intestine	Sannino et al. (2006)
	Polyethylene glycol CMC hydrogel	Urethral bulking agent for hound dogs	Sumner et al. (2012)
	HEC hydrogel	Bulking agent during cell	Sannino et al. (2006)
Biological fuel cells	Bacterial CB hydrogel	Microbial fuel cells	Mashkour et al. (2016), Mashkour et al. (2017), Shi et al. (2014)

## Conclusions

Hydrogels based on biopolymer cellulose are linear polymers with a unique chemical structure having numerous hydrophilic groups enabling them to absorb and retain a huge proportion of water and thus possess a great variety of properties. The combination of versatile physicochemical properties allows hydrogels to have a wide range of industrial and biomedical applications; therefore, attracting a great scientific and industrial interest across the globe. Hence, this review is intended to focus on the promising aspects of cellulose-based hydrogels and their derivatives in biomedical applications as well as in agriculture and other industrial appliances with a meticulous compilation of the available relevant information regarding chemistry and physicochemical properties. In view of these characteristics along with their biocompatibility, biodegradability profile, cellulose-based hydrogels are poised to be an exciting and interesting excipient in the biomedical industry for the present and the future applications in wound dressing, tissue engineering and drug delivery system. The numerous uses of cellulose-based hydrogels even include the development of smart materials, advancement in healthcare and hygienic products as well as in the improvement of agriculture and wastewater treatment. All these explorations indicate that hydrogels based on cellulose hold a great promise for the applications in multidisciplinary areas and may act as a wonder material in the field of biomedical engineering and industrial sectors.
